# Our favorite unproven ideas for future critical care

**DOI:** 10.1186/cc11507

**Published:** 2013-03-12

**Authors:** John J Marini, Jean-Louis Vincent, Paul Wischmeyer, Mervyn Singer, Luciano Gattinoni, Can Ince, Tong Joo Gan

**Affiliations:** 1 University of Minnesota, Regions Hospital MS11203B, 640 Jackson Street, St Paul, MN 55101, USA; 2 Department of Intensive Care, Erasme University Hospital, Université, libre de Bruxelles, Route de Lennik 808, 1070 Brussels, Belgium; 3 Department of Anesthesiology, University of Colorado School of Medicine, 12700 E, 19th Avenue, Box 8602, RC2 P15-7120, Aurora, CO 80045, USA; 4 Department of Intensive Care Medicine, Bloomsbury Institute of Intensive Care Medicine, University College Londonm, Cruxiform Building, Gower Street, London, WC1E 6BT, UK; 5 Dipartmento di Anestia, Rianimazione (Intensiva e Subintensiva) e Terapia del Dolore, Fondazione IRCCS Ca' Granda - Ospedale Maggiore Policlinico, Milan, Italy; 6 Department of Intensive Care, Erasmus MC, University Medical Center Rotterdam,'s-Gravenijkwal 230, 3015 CE Rotterdam; 7 Department of Anesthesiology, Box 3094, Suite 5670B, Duke University Medical Center, Durham, NC 27710, USA

## Abstract

The future of critical care medicine will be shaped not only by the evidence-validated foundations of science, but also by innovations based on unproven and, in many cases, untested concepts and thoughtful visions of scientists and clinicians familiar with the complex problems actually faced in clinical practice. Clinical investigations and trials often lag behind collective experience and impressions, in a well-intentioned and necessary quest to determine the fallacy or validity of ongoing practice. Progress made in this way can be painfully slow, and imperfect theory may prove difficult to challenge. On occasion, an innovative paradigm shift fostered by a novel approach can reorient the forces of academic investigation toward generating an evidence base upon which such concepts and interpretations can find scientific justification. This discussion presents a selected set of ideas to improve the future practice of critical care - each having a defensible rationale, but unconfirmed validity.

## Introduction

While technical innovations and scientific experiments often lead clinical practice, at times clinical science lags behind experience and simply documents what appears to be the fallacy or validity of ongoing practice. Progress made in this way can be slow, and imperfect theory may prove difficult to challenge. What follows is a wide-ranging set of unproven ideas, each having a defensible rationale but unverified worth. The intent of this thought-provoking format is to stimulate thinking and interchange, and perhaps to point toward new directions with potential for productive research, conceptual modification, and eventual applications that could improve care of the critically ill. In what follows, a brief background outlines the rationale for each novel and unproven idea endorsed by the presenter.

## We should retire the stethoscope (J-L Vincent)

### Background

Dating from the days of Laennec, the stethoscope has proven itself a robust and helpful diagnostic instrument [1]. Chief among the instrument's attractive features are its portability, simplicity, ubiquity, low cost, and ability to complement the physical examination by opening an immediate acoustic window into the functional status of the lungs (for example, wheezes, rales) and heart (gallops, murmurs). It is a testimony to past contributions that this tool has not changed fundamentally in more than two centuries. Traditional stethoscopy does have drawbacks, however. Unrecorded sounds heard through the stethoscope are evanescent, the interpretation of the nature, timing, and amplitude of breath sounds is subjective, and high variability between observers is well documented [2]. Although acoustic information can be digitally processed as well as displayed [3], traditional stethoscopic information is descriptive rather than quantitative. The value of auscultation (similar to the unaided physical examination) depends on the skill of the individual and the completeness of reporting [4].

The stethoscope was particularly useful as an aid for physical examination when bedside imaging either was not available or was difficult to interpret. Not only do we now have better quality radiographs, but we also have echocardiography and ultrasound that can be quickly and repeatedly deployed to yield detailed and quantitative imaging information [5]. Bedside computed tomography scanners and even magnetic resonance imagers are not far away from use. A continuous data-stream that yields dynamic functional monitoring may be afforded by emerging technologies such as electrical impedance tomography and acoustic monitoring. By comparison, the venerable stethoscope seems dated and crude [6].

Nonetheless, the no-cost, instantly available stethoscope will continue to have indications, especially when immediate availability and feedback are at a premium. For example, auscultation aids in making sure that an endotracheal tube is in the right track or not placed too far down (decreased breath sounds in one lung). Another use is the classification of very acute respiratory failure: Is the problem tube obstruction? Bronchospasm? Pneumothorax? Hemothorax? While not definitive, auscultation may therefore effectively complement more precise and detailed diagnostic methods [7].

### Idea

Deploy quantitative, recordable, and detailed diagnostic and monitoring technologies at the bedside, while relegating the stethoscope to a lesser screening role for specific, targeted applications.

## De-stressing therapy: blockade of the β-adrenergic system benefits mortality, metabolism, and the immune system (M Singer)

### Background

Our innate responses may be ill adapted to life-threatening illness. One clear possibility is that the native hormonal reaction to such challenges may be noxious, perhaps even accelerating injury processes in the wrong direction [8,9]. Physiological adaptations to catastrophic and therefore unsurvivable conditions are not likely to have evolved naturally. One could even argue that evolution would have selected *against *those genes that would have allowed the organism to experience trauma, infection, or serious acute organ dysfunction. Even the fight or flight response may be excessive.

Overly exuberant upregulation of the β-adrenergic system in response to stresses of various types (for example, cardiovascular crises, sepsis, massive wounding) has been shown to produce necrosis of vital tissues [10]. Published clinical trials demonstrate the potential for β-blockade to reduce myocardial damage following coronary occlusion. Sudden emotional stress may produce dramatic cardiac dysfunction, which persists for weeks afterwards (Takotsubo cardiomyopathy) [11]. β-adrenergic blockade, while contraindicated during an acute attack, may reduce sensitivity to methacholine challenge in the setting of chronic asthmatic disease. Continuation of intense β-stimulation has the opposite effect. β-blockade has experimentally been shown to improve survival in rats challenged with intravenous lipopolysaccharide, and β-blockers are most effective if given before such a challenge [12]. Such observations suggest that interference with hardwired adrenergic responses to physiologic crises (such as severe sepsis) by modulation of their intensity could be a productive therapeutic avenue to pursue.

### Idea

Rather than relying on high-dose catecholamine-based inotropic/vasoactive agents, using noncatechol inotropes (for example, milrinone) in conjunction with closely modulated β-blockade may be a rational approach to supporting the cardiovascular system during the stress of severe sepsis.

## Anabolic/anti-catabolic therapy in critical illness (P Wischmeyer)

### Background

Many patients survive the ICU only to face a protracted period of recovery, permanent disability, or eventual death from immobility. Recent data reveal more than 40% of the 6-month mortality following critical illness occurs after the patient has been discharged from the ICU [13]. Many of these deaths are believed to occur indirectly as a result of catabolism, loss of lean body mass, and lack of adequate physical activity, and to ultimately culminate in profound weakness and an inability to mobilize [14,15]. Many patients report very poor physical function for the entire first year following ICU discharge [14] and these physical dis abilities have been shown to persist for 5 years or longer following the ICU discharge. Survivors of our advanced ICU care are thus often significantly debilitated for months or years afterwards - a process known as ICU-acquired weakness. Many ICU survivors suffer rapid, dramatic loss of lean body mass, causing significant debility that leaves them predisposed to aspiration, pneumonia, and other life-threatening complications [15]. The patient facing the abrupt stresses imposed by critical illness has not had time to evolve appropriate - and in this case adaptive - responses.

The ICU experience undergone by many normal individuals is not unlike the stresses encountered by elite athletes during training and competition. Critical illness progresses through acute, chronic, and recovery phases, each with their different nutritional demands and susceptibilities. Before competition, many of the most successful competitors take anabolic, performance enhancing agents for extended periods that allow them to cope with the stresses of performing exceptionally at the time of their events. Elite strength-training athletes legally utilize high-quality protein (typically 2.0 g/kg/day), glutamine, fish oil (an anti-catabolic), hydroxymethyl butyrate, and creatine. As has been well described in the popular press in sports such as baseball and cycling, many athletes use nonsanctioned androgens (testosterone derivatives) and growth hormone, especially when trying to speed recovery after intense training and injury. Other anabolic hormones, such as insulin and insulin-like growth factor-I, are often stacked together with androgens and growth hormone. Despite risks of unsupervised use, performance enhancers are used because they work.

In recent years, common use of such agents has resulted in world and Olympic records being broken at an unprecedented pace by superhuman athletes. For athletic applications, effective dosing and timing have been worked out. Medical use of these agents, outside the burn unit [16,17], has been quite rare in the ICU setting. Further research is therefore needed to optimize the use of these agents to help our patients recover from the ultimate race - critical illness. In a sense, evolving to survive critical illness and to function again normally would be superhuman, and we should learn from athletes who perform superhuman feats daily to help our patients recover from what Mother Nature would have previously deemed unrecoverable.

### Idea

Use well-timed nutritional and hormonal interventions to improve immunological and neuromuscular responses so as to aid healing and prevent debility. Use high-dose protein (1.5 to 2.0 g/kg/day), exercise (that is, early ambulation of ventilated patients), and hydroxymethyl butyrate during the acute phase, with no potent anabolics. In the later phases, use sufficient calories and high-quality protein, β-blockers, and hydroxymethyl butyrate complemented by oxandrolone and creatine. In the recovery phase, add low doses of growth hormone to this prescription. (Following the acute phase of burns, approaches using β-blockers and oxandrolone have been quite successful, provided that early and adequate protein/calorie delivery is achieved [16,17].) Delivering sufficient calories and protein in the chronic and recovery phases of critical illness is mandatory if oxandrolone, growth hormone, or other anabolic agents are to be both safe and effective.

## After rescue, we should adapt patients to their critical illness physiology (J Marini)

### Background

Critical care evolved from anesthetic approaches and postoperative practice. In elective cases, the sudden trauma of surgery interrupts resting physiology and is addressed by an attempt to restore a normal baseline. In the critical care setting, however, the price of maintaining normality may be injury inflicted by the therapy itself (ventilator-induced lung injury, drug toxicity, consequences of sedation, and so forth). Equally plausible is that our enforcing of normal indicators and protracted use of excessive support may impede the patient's potential to adapt to the abnormal physiology that characterizes chronic critical illness.

Health is characterized by gradual transitions between physiological states, by variation of cardiorespiratory indices, and by continual adaptation to biochemical, environmental, and mechanical stressors. Acute disease, on the contrary, is often characterized by abrupt transitions, failure to adapt to the stressor, and monotonously inflexible physiological patterns [18]. Currently, our treatments rescue very effectively, but when sustained for too long they may frustrate the patient's innate adaptability to stressful but potentially survivable illness. For example, early use of short-term muscle relaxants enables lung-protective strategies and may reduce mortality risk in acute respiratory distress syndrome [19], but when sustained for too long muscle relaxants can cause muscular atrophy and sustained weakness [20]. Because evolution did not provide for appropriate responses to severe acute injuries, an argument can be made that we must moderate the initial assault and response, and then build our capability to respond to lingering challenges over time.

During health, tolerable stresses above the resting baseline that are applied and released build strength, promote tolerance, and remodel tissues. Endurance athletes are a good example of what can be achieved with intermittent exposure to challenging stress. Stress-induced adaptations include enhanced pulmonary oxygen exchange, increased blood volume and hemoglobin concentration, enriched blood flow to skeletal muscle, improved thermal regulation, increased mitochondrial size and density, and increased capillarization of muscular beds. The healthy individual is also extremely adaptable when the physiologic milieu is gradually changed. Important lessons regarding adaptation to abnormal physiology have been learned from protracted exposure to hypoxic inspired gas, both in mountain climbing and in the experimental laboratory [21].

Functionally adaptive response needs some time to develop. For example, after exposure to a thermal stress, heat shock proteins require approximately 18 hours to be expressed to a level that protects against experimental ventilator-induced lung injury and sepsis [22]. Conversely, abrupt and synchronous exposure to the same high temperature may accelerate ventilator-induced lung damage, whereas cooling inhibits its expression [23]. Slowly achieving a given target is protective in other settings. For example, impressive depths of hypoxemia can be tolerated and the dangers of hyperoxia avoided by graded exposure to permissive hypoxemia [24]. The amplitude and timing of these conditioning challenges to the resting baseline and to the intermittent stresses are keys to tolerance and adaptation. Important unanswered questions related to conditioning of the critically ill patient relate to whether injured tissues are capable of stress conditioning and when in the course of the illness is it advisable to time the flip from the full support of rescue to a goal-directed conditioning pattern.

### Idea

A two-stage approach of initial rescue - that emphasizes minimizing demand, full support, gentle transitions, and assuming physiological control - followed by an adaptive phase - characterized by ongoing targeted reduction of vital supports and imposed intermittent stresses and rest periods, akin to the endurance and strength-building programs of athletic training - should be applied. (Such acclimatization and conditioning might be applied to the most common and unchallenged elements of acute care, such as the fraction of inspired oxygen, ventilating pressures, vasopressors, and positioning.) Expressed poetically: 'First shelter the critically ill patient during the storm and then teach the patient to dance in the rain.'

## Limit lung dishomogeneity to prevent lethal injury by the ventilator (L Gattinoni)

### Background

Awareness of the potential for ventilator-caused lung damage has grown over the past 25 years. Clinicians now appreciate that excessive airway pressures are dangerous, particularly when large tidal volumes are employed and insufficient positive end-expiratory pressure is used to keep the lung from experiencing high-pressure tidal opening and collapse cycles [25]. The healthy portions of the injured lungs have relatively normal mechanical properties [26]. A fixed tidal volume crammed into a smaller lung will accentuate stress and strain. The clinically measurable correlates of resting lung volume and stretched lung volume are the functional residual capacity and the combination of the volume attributable to positive end-expiratory pressure and tidal volume [27].

Normal lungs begin to lose their integrity, however, when the gas volume of the given sector exceeds its resting value by a factor of 2.0 to 2.5. Such an extreme expansion of dimensional sizes is rarely seen in uniform lungs in the clinical setting [28]. However, inter dependence amplifies local forces at the interface between open tissue and collapsed tissue, a concept first brought to attention more than four decades ago in the work of Mead, Takashima, and Leith [29]. At these stress riser points, the alveolar-capillary membrane can be stressed sufficiently to break down. When this membrane does break down, the rate of deterioration occurs with impressive speed - presumably because new stress risers are generated and the aerated lung size shrinks. The relatively poor correlation across different patient populations between tidal volume and outcome, even when the latter is adjusted to predicted body weight, perhaps occurs for these reasons. If the number of stress riser points can be minimized, therefore, the lung will be better protected and catastrophic progression of lung injury may be avoided or slowed.

### Idea

Promote uniformity and minimize the generation of stress rising points of heterogeneity by measures such as prone positioning (which reduces the gradient of transalveolar pressure) and recruiting the lung. Monitoring of the injured lung should be conducted with methods that are sensitive to regional heterogeneity - such as computed tomography and possibly electrical impedance tomography, ultrasound, and acoustic mapping - rather than exclusively by the global methods for monitoring lung mechanics and gas exchange in use today.

## Targeting the microcirculation to improve outcome (C Ince)

### Background

Tissue health depends on adequacy of perfusion and oxygenation, not always well reflected by global measures of cardiac output and oxygen extraction. In the setting of sepsis, the endothelium and vasculature are under assault by activated leukocytes, inflammatory mediators, and reactive oxygen species that cause microcirculatory dysfunction in advance of organ failure [30]. A compromised microcirculation is no longer able to regulate blood flow distribution, resulting in functional shunting where the oxygen need of the parenchymal cells is not met by adequate delivery [31]. This condition describes distributive shock, one of the four states of shock defined by Weil and Shubin [32]. Early restoration of perfusion and oxygenation in addition to correction of the imbalances set into motion by endothelial damage, inducible nitric oxide synthase, endothelial nitric oxide synthase, and reactive oxygen species appear to improve outcomes [33]. Measures to reopen the microcirculation include boosting of hematocrit and oxygen transport, increasing the intravascular volume, and control of oxidative stress and inflammatory load.

An extensive body of scientific data collected in an experimental setting documents such complex biochemical interplay. Clinical data demonstrate that early interventions targeting increased global perfusion improve outcomes and lower Sequential Organ Failure Assessment scores. Although the value of such an approach has been established for surgical patients, whether interventional targeting works as successfully for critically ill septic patients remains controversial. The ultimate objective, however, is to oxygenate the microcirculation by improved perfusion with blood that is optimally charged to off-load oxygen by maintaining adequate circulatory dimensions, fine-tuned vasoregulation, and improved plasmatic composition. The latter objective could conceivably be helped by using balanced starches to promote convection and correct hypovolemia. Blood transfusions, in addition to increasing oxygen-carrying capacity, also recruit previously unfilled capillaries and shorten diffusion distances, thereby promoting oxygen transport to tissue [34]. To reverse the consequences of the shock cascade, optimal perfusion must be accomplished at the local microcirculatory level, not simply by improving the performance of the heart and charging the blood with high oxygen content.

In recent years, a variety of techniques that monitor microscopic blood flow and its consequences have been employed in the laboratory setting. These have been brought closer to readiness for clinical implementation [35]. With such technology, the potential efficacy or adverse consequences of drugs and fluids can be monitored in representative tissue beds. The tools used to implement a new paradigm based on controlling inflammation, resuscitating endothelial cells, and modulating reactive oxygen species will require such monitoring instruments.

### Idea

Target the microcirculation as the primary objective. Directly visualize representative microcirculatory beds to monitor progress toward the goal of achieving adequate perfusion and oxygenation. Utilize the drugs and fluids that are effective in oxygenating and perfusing the microcirculation (goal-directed fluid therapy plus blood transfusion), opening the microcirculation (for example, sodium nitrite, a pure nitric oxide donor with potential for releasing nitric oxide from hypoxic red blood cells in compromised tissues), preserving the cellular glycocalyx (for example, vitamin C), and providing potent actions at the microcirculatory level.

## Employ holistic care to take advantage of a missed opportunity (TJ Gan)

### Background

As practiced in advanced industrial societies, western medicine leverages scientifically-based therapies that emphasize artificial procedures and drugs. ICU medicine has been an avid proponent of management that stresses measurement and correction. Traditional eastern practices, on the contrary, embrace a variety of treatments that integrate complementary and alternative measures (CAM) into a less methodically documented holistic approach that conceives of health as a tuned balance of body, mind, and spirit. In recent years, the importance of engaging these missing spheres has also been recognized in the outpatient practices of the advanced industrial world. Diet, exercise, relaxation, massage, prayer, music, and closer involvement of the family - methods that are outside established standards for ICU practice - have increasingly been integrated into intensive care management settings [36-38]. In the outpatient environment, CAM have been recognized as helpful in relieving stress, anxiety, discomfort, restlessness, and insomnia. The existing literature strongly backs the effectiveness of CAM for attenuating pain and anxiety; patients have generally been receptive and ultimately satisfied with their use [39-41]. Demands for inpatient application of CAM are increasingly strong, as they confer a measure of treatment control to the patient and family, and emphasize patient well-being and preferences during their hospital stay. Where implemented in the inpatient setting, CAM methodology has been viewed as clinically effective by patients and physicians alike [38].

The patient with critical illness faces major problems related to sleep, anxiety, and pain [40,41]. These challenges are severe enough to warrant sedation and analgesia in doses that may ultimately compromise progress or cause complications. Recovery may be slow and arduous. CAM methodology is generally free of lasting consequences other than lingering relaxation and reduced pain. Acknowledging a strong recent emphasis on avoiding delirium, a holistic approach is an attractive option to complement our current measures for intensive care [42].

### Idea

Wake patients earlier, use fewer drugs, and incorporate CAM into ICU practice of the alert and semiconscious patient. Use these techniques routinely to accomplish effective sleep, pain relief, and the sense of well-being that will accelerate rehabilitative progress and reduce the consequences of drug-based management.

## Conclusion

The future of critical care medicine will be shaped not only by the evidence-validated foundations of science, but by innovations based on unproven and, in many cases, untested concepts and thoughtful visions of scientists and clinicians familiar with the complex problems actually faced in clinical practice. On occasion, an innovative paradigm shift fostered by a novel idea can reorient the forces of academic investigation to generate an evidence base upon which such concepts and interpretations can find scientific justification. Intriguing as this out-of-the-box thinking may be, however, such visions should not be implemented until supported by a substantial body of basic and clinical research.

## Abbreviations

CAM: complementary and alternative measures.

## Competing interests

The authors declare that they have no competing interests.

## Declarations

This article has been published as part of *Critical Care *Volume 17 Suppl 1, 2013: Future of Critical Care Medicine. The supplement was proposed by Fresenius Kabi based on presentations from the 'Future of critical care medicine (FCCM) 2012: Today's practice and a look to the future' symposium. Articles were commissioned by the journal, were independently prepared by the authors and have been peer reviewed by the journal. The supplement was edited by John Marini, who declares that he has no competing interests. Publication of the supplement was supported by Fresenius Kabi.
